# Snack quality and snack timing are associated with cardiometabolic blood markers: the ZOE PREDICT study

**DOI:** 10.1007/s00394-023-03241-6

**Published:** 2023-09-15

**Authors:** Kate M. Bermingham, Anna May, Francesco Asnicar, Joan Capdevila, Emily R. Leeming, Paul W. Franks, Ana M. Valdes, Jonathan Wolf, George Hadjigeorgiou, Linda M. Delahanty, Nicola Segata, Tim D. Spector, Sarah E. Berry

**Affiliations:** 1 Department of Nutritional Sciences, King’s College London, London, UK; 2 ZOE Ltd, London, UK; 3 Department CIBIO, University of Trento, Trento, Italy; 4 Department of Twin Research and Genetic Epidemiology, King’s College London, London, UK; 5 Department of Clinical Sciences, Lund University, Malmö, Sweden; 6 Department of Nutrition, Harvard TH Chan School of Public Health, Boston, MA USA; 7 School of Medicine, University of Nottingham, Nottingham, UK; 8 Nottingham NIHR Biomedical Research Centre, Nottingham, UK; 9 Diabetes Center, Department of Medicine, Massachusetts General Hospital, Boston, MA USA; 10 Department of Medicine, Harvard Medical School, Boston, MA USA

**Keywords:** Snacking, Quality, Frequency, Quantity, Timing, Cardiometabolic health

## Abstract

**Background:**

Snacking is a common diet behaviour which accounts for a large proportion of daily energy intake, making it a key determinant of diet quality. However, the relationship between snacking frequency, quality and timing with cardiometabolic health remains unclear.

**Design:**

Demography, diet, health (fasting and postprandial cardiometabolic blood and anthropometrics markers) and stool metagenomics data were assessed in the UK PREDICT 1 cohort (*N* = 1002) (NCT03479866). Snacks (foods or drinks consumed between main meals) were self-reported (weighed records) across 2–4 days. Average snacking frequency and quality [snack diet index (SDI)] were determined (*N* = 854 after exclusions). Associations between snacking frequency, quality and timing with cardiometabolic blood and anthropometric markers were assessed using regression models (adjusted for age, sex, BMI, education, physical activity level and main meal quality).

**Results:**

Participants were aged (mean, SD) 46.1 ± 11.9 years, had a mean BMI of 25.6 ± 4.88 kg/m^2^ and were predominantly female (73%). 95% of participants were snackers (≥ 1 snack/day; *n* = 813); mean daily snack intake was 2.28 snacks/day (24 ± 16% of daily calories; 203 ± 170 kcal); and 44% of participants were discordant for meal and snack quality. In snackers, overall snacking frequency and quantity of snack energy were not associated with cardiometabolic risk markers. However, lower snack quality (SDI range 1–11) was associated with higher blood markers, including elevated fasting triglycerides (TG (mmol/L) *β*; – 0.02, *P* = 0.02), postprandial TGs (6hiAUC (mmol/L.s); *β*; – 400, *P* = 0.01), fasting insulin (mIU/L) (*β*; – 0.15, *P* = 0.04), insulin resistance (HOMA-IR; *β*; – 0.04, *P* = 0.04) and hunger (scale 0–100) (*β*; – 0.52, *P* = 0.02) (*P* values non-significant after multiple testing adjustments). Late-evening snacking (≥ 9 pm; 31%) was associated with lower blood markers (HbA1c; 5.54 ± 0.42% vs 5.46 ± 0.28%, glucose 2hiAUC; 8212 ± 5559 vs 7321 ± 4928 mmol/L.s, *P* = 0.01 and TG 6hiAUC; 11,638 ± 8166 vs 9781 ± 6997 mmol/L.s, *P* = 0.01) compared to all other snacking times (HbA1c remained significant after multiple testing).

**Conclusion:**

Snack quality and timing of consumption are simple diet features which may be targeted to improve diet quality, with potential health benefits.

**Clinical trial registry number and website:**

NCT03479866, https://clinicaltrials.gov/ct2/show/NCT03479866?term=NCT03479866&draw=2&rank=1

**Supplementary Information:**

The online version contains supplementary material available at 10.1007/s00394-023-03241-6.

## Introduction

Snacking can account for a large proportion of daily energy intake, making it a key determinant of diet quality [1, 2]. Despite the large energy contribution of snacking to diets, and the huge heterogeneity in the healthfulness of snacks consumed, there is very little research on the impact of snacking frequency, nutritional quality and timing on multiple cardiometabolic blood and anthropometric markers. Further, the lack of clear consensus on snacking advice from public health authorities [3] and conflicting evidence regarding the impact of snacking on weight and health is likely due to the challenges in defining snacks (frequency, quantity and quality) and lack of consideration for background diet and timing of snack consumption.

Snacks can be defined based on the time of day when consumed [4, 5], food type or amount [6], by self-reporting [7–9] or by some combination of these factors [1]. Internationally, the most popular snacks include poor quality (heavily salted, sweetened and/or high-fat) foods such as chips, desserts and sugar-sweetened beverages [2, 10, 11]. Several studies suggest that snacking behaviour can benefit health by distributing energy and nutrients across multiple eating occasions in a day [11] or through beneficial food-specific components, with favourable impacts on lipid profiles (cholesterol and TG concentrations), glucose homeostasis [12], blood pressure [13] body weight [14, 15], gut microbiome composition [16, 17] and cardiovascular disease mortalities [18], possibly because snacking may suppress hunger and appetite. Research suggests that pre-existing health status and snack quality may influence snacking’s impact on health [19, 20]. For example, regular snacking on whole almonds significantly improved endothelial function and lowered LDL cholesterol in individuals with higher CVD risk [19] and compared to a popular snack (i.e. sweet biscuits) improves glycaemic control and energy intake [12]. Snacking was also negatively associated with body fat in lean individuals (BMI < 25 kg/m^2^) but positively associated with waist circumference and subcutaneous fat in people who are overweight or living with obesity [20]. Furthermore, healthy snack choices may mitigate unfavourable associations, as people who were overweight or living with obesity consumed more unhealthful snacks compared to healthy weight participants [20].

The inconsistencies of snacking research render the impact of snacking on health unclear. Further investigation of snacking behaviour is warranted, specifically, whether snacking frequency, quality and/or timing are key determinants of cardiometabolic health [10]. We leveraged the densely phenotyped ZOE PREDICT 1 cohort, containing high-resolution weighed logged diet records, to (1) characterise free-living snack intakes; (2) explore the relationship between snacking frequency, quality and timing on cardiometabolic blood and anthropometric measures; and (3) investigate whether snacking frequency or quality is associated with the gut microbiome.

## Subjects and methods

The *ZOE* PREDICT 1 study is a diet intervention study, conducted between 5 June 2018 and 8 May 2019, examining diet-cardiometabolic interactions, including *N* = 1,002 from the UK. The full protocol [21] and primary outcomes are reported elsewhere [22]. In brief, the primary cohort was recruited at St Thomas’ Hospital (London, UK) from the TwinsUK cohort, an ongoing research cohort, and through online advertising. Healthy UK adults including non-twins, monozygotic twins and dizygotic twins were enrolled and completed baseline clinic measurements. The study consisted of a 1 day clinical visit (day 1) at baseline followed by a 13-day at-home period. Study participants were healthy individuals aged 18–65 years who were able to provide written informed consent. Criteria used to assess eligibility and exclusion criteria are listed elsewhere [21]. Ethical approval was granted by the London-Hampstead Research Ethics Committee (approval no. 18/LO/0663) and Integrated Research Application System (no. 236407). Detailed information, including the food logging mobile study app used to assess snacking and the health and lifestyle questionnaire data collected, is available in the online protocol [21]. The informed consent and ethical committee approvals covered all analysis reported in the current study in addition to the key primary outcomes [22]. The trial is registered at ClinicalTrials.gov (NCT03479866) and was run in accordance with the Declaration of Helsinki and good clinical practice.

This secondary analysis is a cross-sectional analysis of the baseline data and weighted logged diet data obtained as part of the original intervention trial. Out of the *N* = 1002 initially enrolled participants, *n* = 967 successfully completed the study. Additional exclusion criteria applied for this secondary analysis included participants who travelled across time zones during the free-living period (*n* = 13) and those with < 2 days of free-living weighted logged diet records post cleaning (*n* = 100). The final cohort included in this analysis was *n* = 854 (Supplementary Fig. 1, CONSORT diagram).

### Diet data

In the ZOE PREDICT 1 cohort, participants recorded all diet intakes during the entire study period on the specialised ZOE study app, yielding comprehensive records of timed intakes. Participants were trained to accurately record ad libitum diet intake using photographs, product barcodes, product-specific portion sizes and weighed intakes using digital scales. Data logged into the study app were uploaded onto a digital dashboard in real time and assessed for logging accuracy and study compliance by study staff (Criteria for accuracy assessment were previously described in Berry et al. [22]). Any uncertainties were clarified actively with the participant through the app’s messaging system or via phone while on the study. Nutrient composition of weighted logged foods was obtained from the McCance and Widdowson Food and Nutrient database [23]. For branded food items, nutrient composition was collated from common supermarket websites.

Participants self-reported meal type (i.e. snack, breakfast, lunch, dinner or drink) when they logged food items. An eating occasion was defined as any occasion where a food or beverage was separated in time from the preceding and succeeding eating occasion by 30 min. Foods or drinks consumed within the same 30 min window of a meal were considered part of the meal. Where snacks were consumed with a main meal, they were relabelled as a component of the main meal. After aggregating foods and drinks into eating occasions, 86% of eating occasions contained a single meal type (i.e. snack only) and 13% contained multiple meal types (i.e. breakfast and snack). Calorie and nutrient information were summed within each eating occasion.

During the study home-phase, participants consumed multiple standardised test meals over a 9–11 day period, differing in macronutrient composition and order (See online protocol for further detail [21]). The study had a total of 3 test meal protocol groups, two of these protocol groups consumed meals on days 1–12, and the third group consumed meals on days 1–10. Study days where no standardised meals were consumed (range 2–4 days per participant based on protocol group) were classified as “free living”, and main meals and snacks were examined on these days only. On these days exclusions included (1) foods with unidentifiable names and no nutrient information due to logging error, (2) foods with implausible quantities (> 2000 g), (3) alcoholic beverages, (4) days where caloric intakes were outside gender specific cut-offs (females; 500–5000 kcal, males; 500–8000 kcal) and 5) participants with < 2 free-living days of weighted logged diet records. Alcoholic beverages were not classified as snacks as we did not want to capture the effects of alcohol with snacking frequency. Instead habitual alcohol intake, measured using the European Prospective Investigation into Cancer and Nutrition (EPIC) food frequency questionnaire (FFQ), was tested as a covariate in our analysis. A sensitivity analysis showed alcohol consumption did not significantly contribute to the model so was removed as a covariate for the final analysis.

### Assessment of snacking

Snacks were defined as foods or drinks consumed between meals. Snacking events contained (1) a single food type, e.g. apple, or (2) multiple food types, e.g. apple, nut butter and coffee. For the single food snack type, drinks ≤ 50 kcal were excluded to ensure low-calorie drinks did not inflate snacking frequency. To determine the average snack frequency, the number of snacking occasions per participant per day was summed and the number of snacking occasions was averaged across all free-living days. Snacks were mapped onto a “Food Tree” consisting of a database of nutrient information arranged according to a hierarchical tree structure as follows: level 1 (9 food groups); level 2 (52 food groups); and level 3 (195 food groups). These foods were mapped on to the Composition of Foods Integrated Datase**t** (CoFID) for the UK [24] using food categories or subgroup codes. The focus of this categorization system is on grouping similar foods and beverages together based on usage and nutrient content. Snack foods were categorised and fell into 138 food groups within the Food Tree level 3. The most frequently consumed snack foods were classified as those with ≥ 100 logged events, and their contribution to total daily energy intake was calculated.

### Diet quality scores

In order to capture the overall quality of the snacks an individual consumes, we created a snack diet index (SDI) in snackers (those consuming ≥ 1 snack/day). This used an adapted version of the plant-based diet index [25] including animal products and assigned positive scores to healthful snack foods and reverse scores to unhealthful snack foods (Supplementary Table 1) in line with our previous work [26] and epidemiological studies [27–33]. To assess the processing level of snacks, we also applied the NOVA classification [34] to all snacks foods, and classification was carried out by two independent dietitians. In order to capture the quality of the remainder of the diet (excluding snacks), the remainder of the weighted logged diet data was used to calculate the original plant-based diet index (oPDI), which was used as a measure of main meal quality. Participants also completed an adapted EPIC-FFQ to measure habitual diet [35]. Nutrient composition of FFQ foods was determined using FETA software [36] to calculate macro- and micronutrient intakes. Submitted FFQs were excluded if more than 10 food items were left unanswered, or if the total energy intake estimate derived from FFQ as a ratio of the subject’s estimated basal metabolic rate (determined by the Harris–Benedict equation [37]) was more than 2 SD outside the mean of this ratio (< 0·52 or > 2·58) as previously described [22]. Habitual intakes of the 147 foods were used to calculate the oPDI, which was used as a measure of habitual diet quality [25].

### Hunger ratings

Participants reported their hunger levels on a visual analogue scale daily. App notifications appeared at *t* = 0 (time of logging) and regular intervals (+ 0.5, + 1.5, + 2.5 h) following the logging of a breakfast, lunch or dinner meal. However, variable numbers of hunger ratings per day per participant occurred due to missed ratings. An average study hunger score was calculated using all ratings throughout the study period, in participants with ≥ 7 days of hunger ratings.

### Activity levels

Physical activity was self-reported, captured using the following question “In the past year, how frequently have you typically engaged in physical exercises that raise your heart rate *and* last for 20 min at a time?”.

### Gut microbiome

Stool samples were collected by participants at home prior to the clinic visit using an EasySampler collection kit (ALPCO) and put into faecal collection tubes containing DNA/RNA Shield buffer (Zymo Research). A total of *N* = 1001 stool samples were collected and processed for shotgun metagenomic sequencing of which *n* = 854 with snacking data were included in this secondary analysis. In brief, DNA was isolated by QIAGEN Genomic Services using the DNeasy 96 PowerSoil Pro kit and libraries were prepared for 300-bp paired-end reads and sequenced using the Illumina NovaSeq 6000 platform with the S4 flowcell according to the manufacturer’s protocols. The microbiome methodology including DNA extraction and sequencing, metagenome quality and pre-processing, microbiome taxonomic and functional potential profiling and metagenomic assembly have been previously described [21] and have been updated to MetaPhlAn 4.0 [38] for taxonomic profiling of species-level genome bins (SGBs) [39], and to HUMAnN 3.5 for functional profiling [40].

### Cardiometabolic blood and anthropometric measures

The methods for anthropometric and biochemical measures are described in full elsewhere [21, 22]. At baseline (day 1), participants arrived fasted and were given a standardized metabolic challenge meal for breakfast (0 h; 86 g carbohydrate, 53 g fat, 16 g protein, as a muffin and milkshake) and a test lunch (4 h; 71 g carbohydrate, 22 g fat, 10 g protein, as a muffin). The fat was high-oleic sunflower oil: 85% oleic acid (18:1n-9) and 8% linoleic acid (18:2n-6). Fasting and postprandial (0–6 h) venous blood was collected. Briefly, participants were cannulated, and venous blood was collected at fasting (before the test breakfast) and at 9 postprandial time points (15, 30, 60, 120, 180, 240, 270, 300 and 360 min). Plasma glucose and insulin were quantified at all time points, and serum TG was quantified at hourly intervals only. Fasting samples were analysed for lipid profile, including total cholesterol and low density lipoprotein cholesterol (LDL-C). Assays were performed by Affinity Biomarkers Labs. 2 h iAUC (glucose and insulin) and 6 h iAUC (TG) were calculated for analysis. GlycA concentrations were quantified at 3 time points from venous blood at fasting, 4 h postprandial and 6 h postprandial. GlycA was measured using a high-throughput NMR metabolomics (Nightingale Health) 2016 panel, with a CV of 1.1% [41]. Insulin sensitivity (HOMA-IR) and 10-year ASCVD risk, per the 2019 ACC/AHA guidelines [42], were calculated. Visceral fat mass (VFM) was measured using DXA-based visceral fat measurements [43]. Anthropometric measurements including waist and hip circumference were made. Blood pressure was measured in triplicate by trained nurses and the average of the second and third measurements used.

### Statistical analysis

Data analysis was performed using Python 3.8.3 edition (Pandas 1.3.3, statsmodel 0.13.2, scipy 1.7.1). Descriptive characteristics of the cohort and diet intakes were examined. The relationship between snacking frequency, quantity from energy and timing with the cardiometabolic health outcomes was assessed using analysis of covariances (ANCOVA). Linear regression analysis was used to examine the associations between snacking quality (SDI) with the cardiometabolic blood and anthropometric measures. All analyses (ANCOVA and linear models) were adjusted for age, sex, BMI, physical activity, education and main meal quality (oPDI). Participants were stratified across sexes (males and females), age groups (18–35 years, 36–45 years and 46–65 years) and physical activity levels (< 1/week, 1–4/week and ≥ 5/week), and differences in energy intake from snacks between the groups were examined using ANCOVA for the three variables separately. Participants were also stratified across BMI categories (healthy weight; < 25 kg/m^2^ and overweight/obese), and differences in snacking frequency and quality were tested. Spearman’s correlations were used to examine associations between overall diet quality, main meal quality and snack quality. Top and bottom quintiles based on snacking quality (SDI) were selected within frequent snackers (≥ 2 snacks/day), and differences between quintile groups and non-snackers were tested using ANCOVAs. The distribution of snack times over the day was visually examined to identify natural cut-points. Based on this approach, four major snack time periods were identified including: morning (< 12:00 pm), afternoon (12:00–6:00 pm), evening (> 6:00 pm) and grazers (participants with no clear peak). Participants were classified into snack time groups based on when they consumed > 50% of total calories from snacks within these four periods. Participants that consumed any snacks ≥ 9 p.m. were classified as late-evening snackers.

The analysis of the microbiome data was undertaken using a machine learning framework, the same as developed in Asnicar et al. [26]. In brief, prediction and classification of snacking frequency and snacking quality are based on random forest algorithms for both regression and classification tasks. A cross-validation approach was implemented with 100 bootstrap iterations and an 80/20 random split into training and testing folds. To avoid overfitting because of the twins’ population, any twin from the training set was removed if their twin pair was present in the same testing set. For the regression task, we trained a random forest regressor to learn the feature to predict and a simple linear regression to calibrate the predicted values based on the range of the training folds. For the classification task, we trained a random forest classifier to be able to discriminate between the two classes. For continuous values, the bottom and top quartiles were used to define the two classes to discriminate. As microbial features, we considered species-level only taxonomic relative abundance calculated as described above. The continuous values of snacking data were divided into two classes [snacking frequency groups (non-snackers vs frequent snackers (≥ 2 snack/day) and snacking quality groups (frequent snackers with high SDI vs low SDI)].

To ensure the impact of snacking quality on the microbiome was independent of the whole diet, we defined 100 random subsets each of 200 individuals containing 100 good and 100 bad snackers (according to their SDI score). The requirements for identifying these 100 random subsets were that they should not be statistically significantly different between their meal quality scores (according to the Mann–Whitney *U* test) and with a maximum overlap of 50% of individuals with other subsets (Jaccard < 0.5).

## Results

**Fig. 1:**
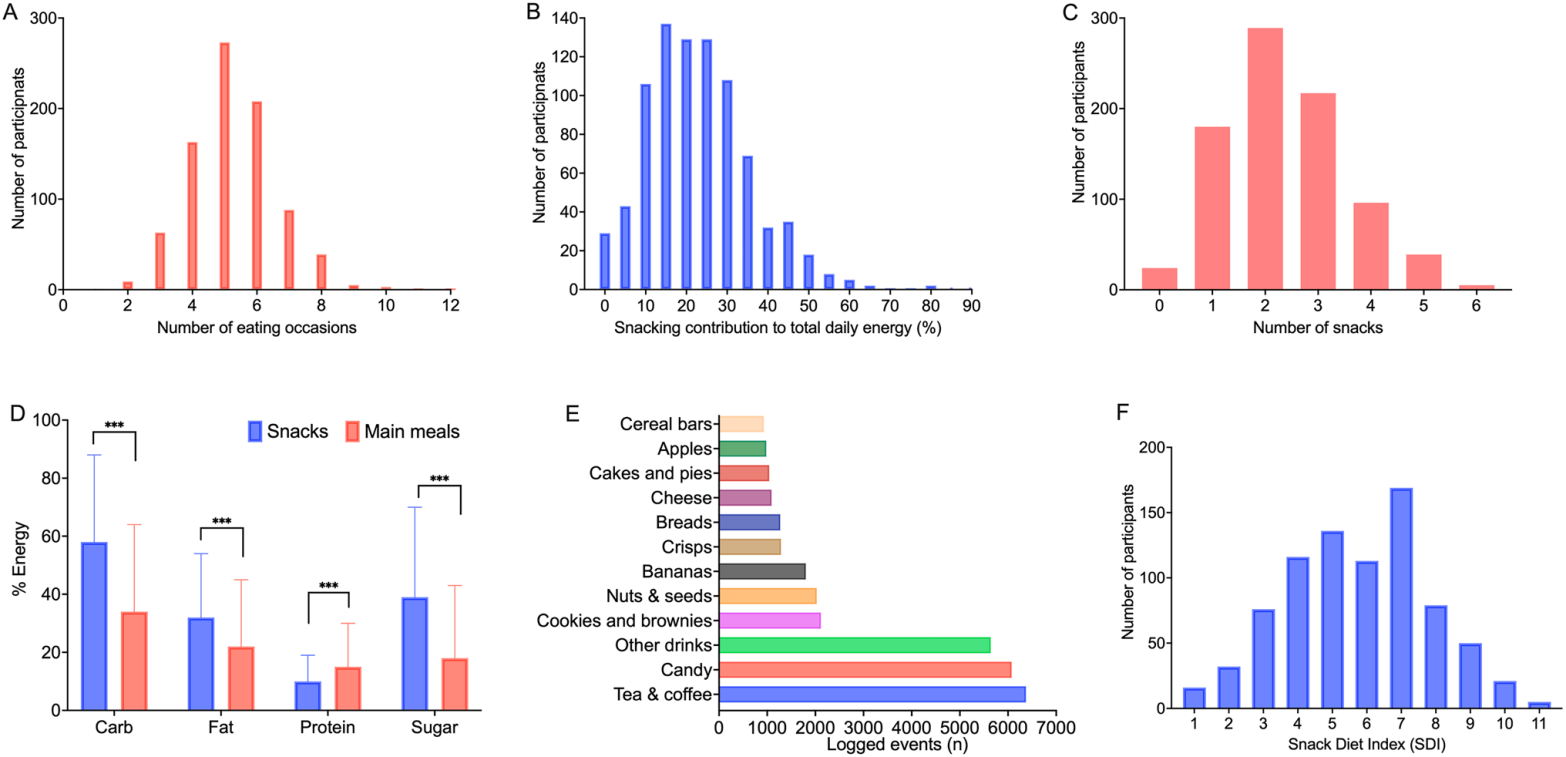
Snacking habits in the PREDICT 1 cohort (*n* = 854). Distribution of **A** total eating occasions (self-reported meals and snacks), **B** percentage of total daily energy from snacks in the total cohort (snackers and non-snackers), **C** snacking frequency, **D** contribution of nutrients (carbohydrates, fat, protein and sugar) from snacks and main meals to total daily energy intake, **E** most frequently consumed foods and drinks logged in snacking events, **F** distribution of the snack quality index (SDI)

Participants were aged (mean, SD) 46.1 ± 11.9 years, had a mean BMI of 25.6 ± 4.88 kg/m^2^ and were predominantly female (73%) (Table 1). Mean daily energy intake was 2366 ± 864 kcal in males and 1864 ± 663 kcal in females. Multiple eating occasions occurred throughout the day (Fig. 1A) with dinner contributing greatest to energy intakes (598 ± 374 kcal), followed by lunch (510 ± 301 kcal), breakfast (380 ± 232 kcal) and snacks (191 ± 163 kcal). These meals contributed on average 35%, 28%, 21% and 24% to total daily energy intake, respectively [average of proportion of calories from each eating event (consumers only)] (Fig. 1B distribution of energy from snacks).

**Table 1:** Characteristics of the cohort

	*n* (%)	Mean (SD)
Age (year)	854	46.1 (11.9)
BMI (kg/m^2^)	854	25.6 (4.88)
Sex (female, %)	854	73
Smoker (currently yes, %)	830	5
Ethnicity		
White (%)	768 (90)	
Asian or Asian British (%)	11 (1)	
Black, African Caribbean or Black British (%)	19 (2)	
Mixed or multiple ethnic groups (%)	20 (2)	
Other ethnic group (%)	7 (1)	
Unknown (%)	29 (3)	
Mean daily energy		
Total cohort (kcal)	854	1995 (754)
Males (kcal)	230	2366 (864)
Females (kcal)	624	1864 (663)
Contribution of nutrients to daily energy		
Carbohydrate (%)	854	58 (31)
Fat (%)	854	32 (23)
Protein (%)	854	10 (9)
Sugar (%)	854	39 (31)
Contribution of snacks to total daily energy (%)	831	24 (16)
Daily calorie intake by snacking frequency		
0 snacks (kcal)	41 (5)	1933 (805)
1 snack/d (kcal)	163 (19)	2292 (936)
2 snacks/s (kcal)	405 (47)	2355 (741)
> 2 snacks/d (kcal)	245 (29)	2517 (697)

### Diet and snacking in the ZOE PREDICT 1 cohort

The average daily snack intakes in people who snack (95% of the cohort) were 2.28 snacks/day (95% CI 2.21–2.35) (Fig. 1C) [total cohort (snackers and non-snackers); 2.18 (95% CI 2.11–2.25)]; 19% consumed 1 snack/d (*n* = 163), 47% 2 snacks/day (*n* = 405) and 29% > 2 snacks/day (*n* = 245). A linear trend was observed for the mean proportion of total daily calories from snacks across snacking frequency groups (1 snack/day; 16 ± 13%, 2 snacks/day; 22 ± 15% 3 snacks/day; 31 ± 16%) (*P* < 0.001). Total daily energy intake increased with increased snacking frequency (*P* < 0.001). Further, snacking frequency and energy from snacks (quantity) were positively associated with habitual intakes of sugar (% EI) (*P* = 0.03) and fat (% EI) (*P* = 0.01) respectively (EPIC-FFQ). The macronutrient contribution to the diet (as % energy) was different for snacks versus main meals (all non-snack energy); snacks were higher in carbohydrates (58% vs 34%), fat (32% vs 22%) and sugar (39% vs 17%) and lower in protein (10% vs 15%) (Fig. 1D).

The most popular foods consumed as snacks included drinks (milk, tea, coffee, fruit drinks), candy, cookies and brownies, nuts and seeds, fruits (apples, bananas, citrus fruits), crisps, bread, cheese and butter, cakes and pies and granola or cereal bars (Fig. 1E) (Supplementary Table 2). Those with the greatest contribution to total daily energy intake were cakes and pies (14%EI), breakfast cereals (13%EI), ice cream and frozen dairy desserts (12%EI), donuts and pastries (12%EI), candy (11%EI), cookies and brownies (11%EI), nuts and seeds (11%EI) and corn snacks, i.e. chips, puffs (11%EI) (Supplementary Table 2). Energy from snacking was broadly similar across sexes (males; 23 ± 16% and females; 24 ± 16%), age groups (18–35 years; 23 ± 14%, 36–45 years; 26 ± 16%, 46–65 years; 24 ± 16%) and physical activity levels (< 1/week; 26 ± 17%, 1–4/week; 24 ± 16% and ≥ 5/week; 24 ± 16%) (*P* > 0.05 for all).

### Snacking quality versus habitual diet

Average snacking quality (SDI; lower scores are indicative of poorer snack quality) was 5.73 ± 2.09, range; 1–11, and IQR; 4–7 (Fig. 1F). Snacking quality was weakly positively associated with habitual diet quality (baseline EPIC-FFQ) (*r*; 0.06, *p* = 0.08), as was quality of food-only snacks (*r*; 0.05, *p* = 0.18). Associations between snacking quality and main meal quality (oPDI, uPDI, hPDI) were low (*r*; 0.16–0.26), highlighting the discordance between these 2 behaviours and their capturing of different dietary attributes (Supplementary Fig. 2). We found that 44% of participants were discordant for meal and snack quality [falling into top quartiles of snack quality (Q3 and Q4) and bottom of quartiles for main meal quality (Q1 and Q2)]; 18% of participants had good snack quality and poor main meal quality, whereas 26% had good main meal quality and poor snack quality. When stratified across BMI groups (healthy weight; < 25 kg/m^2^ (*n* = 435) and overweight/obese; ≥ 25 kg/m^2^ (*n* = 378)), there were no differences in snacking frequency (2.21 ± 1.24 vs 2.15 ± 1.24 snacks/day, *P* = 0.43) or snacking quality (SDI; 5.77 ± 2.02 vs 5.69 ± 2.16, *P* = 0.58).

### Snacking frequency and energy quantity are not associated with cardiometabolic health

Across the snacking frequency groups (0, 1, 2 and > 2 snacks/day), there were no differences in cardiometabolic blood or anthropometric markers including anthropometric traits (height, weight, BMI, visceral fat, waist-to-hip ratio), or fasting and postprandial blood markers (see Supplementary Table 3) (all adjusted for age, sex, BMI, physical activity level and main meal quality). Similarly, we saw no differences in the same cardiometabolic blood or anthropometric markers across the quartiles of quantity of energy from snacks (Q1 < 25, Q2; 25–50, Q3; 50–75, Q4 > 75%). These trends did not change when examining food-only snacking.

### Snacking quality is associated with cardiometabolic health

Participants consumed on average 74% of their snacking calories and 18% of their total daily calories from unhealthful foods. An inverse association was found between snacking frequency and quality (1 snack/d; 6.29 ± 1.67, 2 snack/day; 5.81 ± 2.11 and > 2 snacks/day; 5.23 ± 2.19, *P* < 0.001). Lower snacking quality was associated with higher cardiometabolic blood markers (Supplementary Table 4) including fasting TG (mmol/L) (*β*; – 0.02, *P* = 0.02), postprandial TG (6hiAUC mmol/l/s; *β*; – 400, *P* = 0.01), insulin (mU/L) (*β*; – 0.15, *P* = 0.04) and insulin resistance (HOMA-IR; *β*; – 0.04, P = 0.04)). Participants with lower snack quality also reported greater levels of hunger (*β*; – 0.52,* P* = 0.02). Significance was lost after adjustment for multiple testing (FDR adjustment). When we analysed food-only snacks (excluding drinks), the same health outcomes were significant (TG, postprandial TG, insulin and HOMA-IR) as well as HbA1c levels (*P* < 0.05). After stratification for BMI (healthy vs overweight/obese), higher snacking quality was favourably associated with lower TG, insulin, HOMA-IR, postprandial TG concentrations and lower hunger in individuals living with overweight/obesity, while only GlycA was positively associated with higher snack quality in healthy weight individuals (Supplementary Table 5) (rendered non-significant after multiple testing).

A sensitivity analysis was performed where only 2 free-living days were selected for all participants, and the associations between snacking quality (SDI) with the cardiometabolic blood and anthropometric measures were repeated. The relationships between snack quality and TG, insulin and HOMA-IR persisted (Supplementary Table 6).

### Minimally processed snacking is associated with cardiometabolic health

The SDI was inversely correlated with ultra-processed snacks (% of snacking energy from NOVA 4) (rho; -0.41, *P* < 0.001) and positively correlated with unprocessed and minimally processed snacks (% of snacking energy from NOVA 1) (rho; 0.30, *P* < 0.001). Participants who snack with the highest (Q5, *n* = 157) versus lowest (Q1, *n* = 158) intakes of unprocessed and minimally processed snacks (NOVA 1) had lower weight (*P* = 0.02), BMI (*P* = 0.03), visceral fat mass (*P* < 0.001), fasting glucose (*P* = 0.03), fasting TG (*P* < 0.001), GlycA (*P* = 0.03) and postprandial TG concentrations (*P* = 0.049) (adjusted for age, sex, BMI, physical activity, education and main meal quality). Visceral fat mass and fasting TG remained significantly different after adjustment for multiple testing (*P* < 0.001 for both). Interestingly, the single difference in health outcomes between top and bottom quintiles of ultra-processed (NOVA 4) snack intakes was fasting TG concentrations (Supplementary Table 7).

### High-quality snacking versus low-quality snacking

Frequently snacking on high-quality foods (SDI Q1; ≥ 7, *n* = 49) was associated with favourable body composition, compared to both non-snackers and low-diet quality frequent snackers (SDI Q5; ≤ 3, *n* = 49) (adjusted for age, sex, BMI, physical activity, education and main meal quality); both BMI and visceral fat mass were higher in non-snackers compared to high-quality snackers (BMI; 25.9 ± 5.51 kg/m^2^ vs 22.0 ± 3.04 kg/m^2^ and visceral fat mass; 583 ± 309 g vs 458 ± 256 g) (**Supplementary Table 8**). Additionally, body composition (weight and BMI) was favourable in high-quality snackers versus low-quality snackers (*P* < 0.05 adjusted for covariates); non-significant after multiple testing adjustments). HbA1c concentrations were also higher in low-quality snackers compared to non-snackers (5.51 ± 0.27% vs 5.41 ± 0.24%). In frequent snackers, the most common snack for individuals with high snack quality (Q5) was nuts and seeds (12%EI), whereas for low-quality snackers (Q1) it was cakes and pies (16%EI).

### The relationship between timing of snacks and cardiometabolic health

**Fig. 2:**
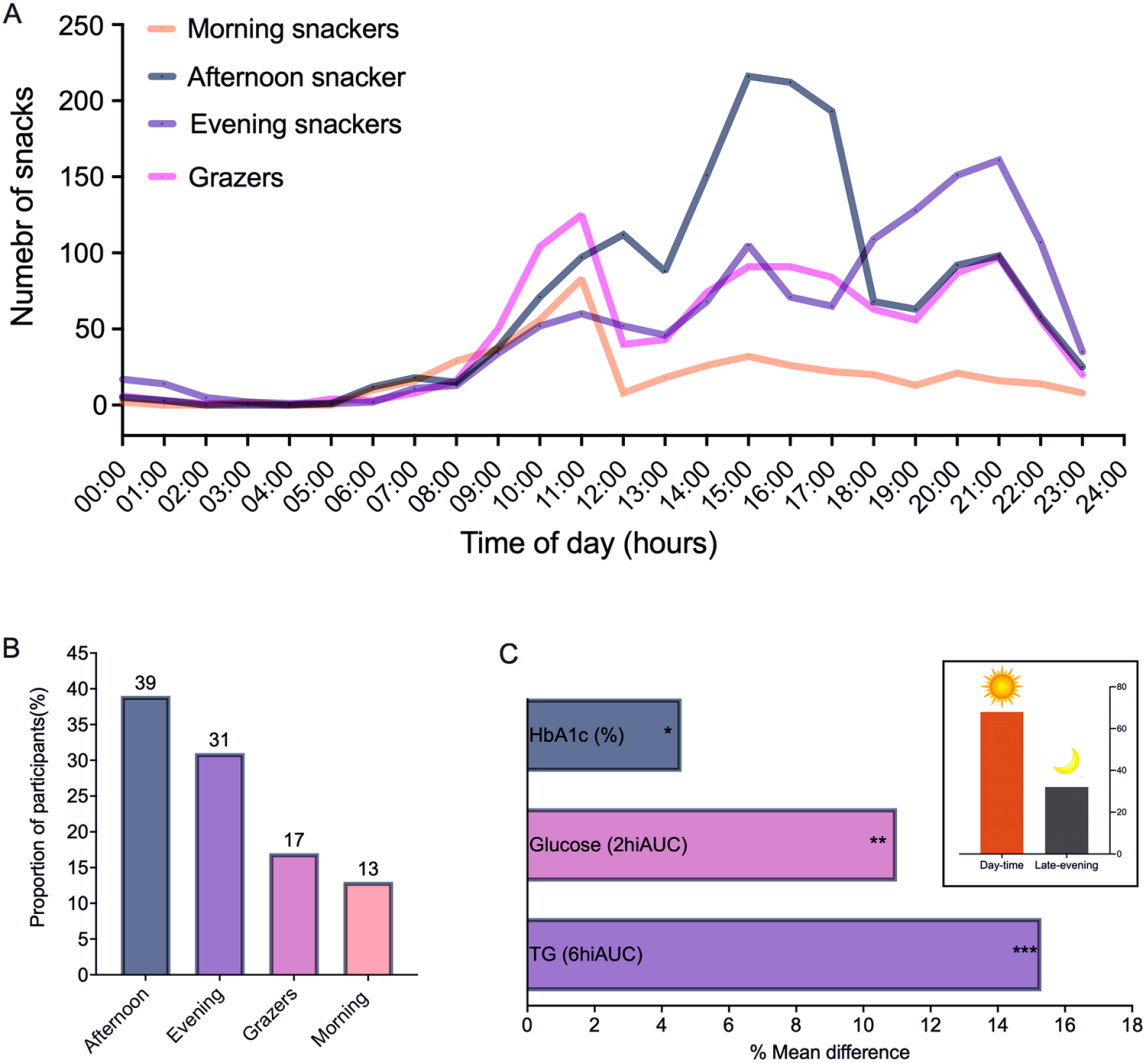
Patterns of snacking across the day. **A** Number of snacks consumed across the day by snacking pattern type (all snackers *n* = 813). **B** Proportion of participants across snacking pattern types, i.e. morning (< 12 pm) (*n* = 106), afternoon (12-6 pm) (*n* = 317), evening (> 6 pm) (*n* = 252) and grazer (*n* = 138) type. **C** Proportion of participants that were late-evening (*n* = 259) or day-time (*n* = 554) snackers and differential markers of health between late-evening and day-time snackers

Four clear temporal snacking patterns, capturing the timing and frequency of snack intake across the day, were evident (Fig. 2A**)**; 13% of participants were morning snackers (≥ 50% of calories from snacks before 12 pm), 39% afternoon snackers (≥ 50% of calories from snacks between 12 and 6 pm), 31% evening snackers (≥ 50% of calories from snacks after 6 pm) and 17% were grazers (participants with no snacking peak) (Fig. 2B). Temporal patterns were associated with snacking quality and quantity; morning snackers had higher SDI (6.42 ± 1.94) and lower energy intake (20 ± 15%) versus evening (SDI; 5.74 ± 2.09 and energy; 25 ± 17%), afternoon (SDI; 5.64 ± 2.06 and energy; 23 ± 15%) and grazing (SDI; 5.33 ± 2.12 and energy; 28 ± 15%) snackers (*P* < 0.001).

Furthermore, individuals who snack after 9 pm (32%), classified as late-evening snackers, had higher HbA1c concentrations (5.54 ± 0.42% vs 5.46 ± 0.28%, *P* < 0.001), postprandial glucose (2hiAUC; 8212 ± 5559 mmol/L.s vs 7321 ± 4928 mmol/L.s, *P* = 0.01), and postprandial TG (6hiAUC 11638 ± 8166 vs 9781 ± 6997, P = 0.01) compared to all other snackers (day-time snackers) (Fig. 2C) (*P* < 0.05 adjusted for covariates); HbA1c remained significant after multiple testing adjustments (*P* = 0.008)) (Supplementary Table 9). Late-evening snackers with poorer snack quality (SDI) had worse fasting TG (*β*: – 0.039, *P* = 0.03) and postprandial TG (*β*: – 751, P = 0.008) than late-evening snackers with higher SDI. When snackers were matched for the first eating occasion time (*n* = 531), late-evening snackers (*n* = 164) had worse Hba1c and postprandial glucose, suggesting that snacking may be detrimental if it reduces the overnight fasting interval (*P* < 0.05 adjusted for covariates); HbA1c remained significant after multiple testing adjustments (*P* < 0.001)) (Supplementary Table 9).

### The relationship between snacking and the gut microbiome

The microbiome composition differentiated individuals based on their snacking quality (AUC = 0.617). As the frequency of snacking might also be related to the quality of the snack, we tested whether the gut microbiome was able to discriminate participants that snack rarely versus those that snack regularly, but we did not see an association (AUC = 0.521). We matched participants for meal quality (see Methods), and the average AUC was reduced to 0.555. This suggests overall diet may have stronger effects on the microbiome than snaking alone.

## Discussion

This research demonstrates snacking is a common dietary behaviour in a UK population accounting for 24% of daily energy intake. The relationship between snacking quality and main meal quality was low, highlighting the discordance between these two behaviours and their capturing of different dietary attributes suggesting that snacking may be a key diet strategy to improve health. We address unanswered questions relating to the importance of snacking frequency, quantity, quality and timing to cardiometabolic health, taking into account the whole day’s diet. Contrary to public perception, we find that the act of snacking, in terms of both frequency and quantity of energy from snacks, was not associated with unfavourable cardiometabolic blood or anthropometric markers. Instead, we observed that snack quality matters and is associated with favourable lipemic and insulinemic responses, as well as decreased hunger. Frequent high-quality snack intake was also associated with favourable weight and BMI compared to non-snackers and frequent low-quality snackers. We identified four temporal snacking patterns based on the time of day and show late snacking is associated with unfavourable outcomes, potentially due to a reduced overnight fasting interval. These findings support the view that snacking on high-quality foods earlier in the day can be part of a healthy lifestyle.

High-quality snacks include whole fresh fruit and vegetables, nuts and seeds which are typically high in fibre and other healthful food components, while retaining their food matrix structure. These foods play roles in mediating hunger and appetite [44], glycaemic control [12, 45] and other cardiometabolic risk markers [19]. For example, the ATTIS study found that regular snacking on whole almonds significantly improved endothelial function and lowered LDL cholesterol in individuals with higher CVD risk [19]. High-quality snack foods are less processed, and to the best of our knowledge this is the first study linking snacking quality by processing level (NOVA classification) to health. UK consumption of ultra-processed foods is estimated to account for 50% of energy [46], and energy-dense foods such as confectionery, packaged savoury snacks and sugar-sweetened beverages are main constituents of snacks [47]. Our research supports increasing intakes of high quality, minimally processed snacks given their positive impact on health [47].

Circadian regulation of metabolic pathways implies that foods may be metabolised differently throughout the day and evidence suggests late snacking is associated with adverse health [48–50]. Late snacking has been shown to significantly lower lipid oxidation compared to an equivalent meal consumed at breakfast, independent of the overnight fast duration [49]. In Japanese individuals living with type-2 diabetes, frequent after-dinner snacking (≥ 3 times/week) was associated with higher BMI and HbA1c levels [50]. Observational data suggest that evening snacking is associated with intakes of energy-dense foods, high in fat and sugar, and our findings support the unfavourable effects of late snacking on health and demonstrates this relationship may be attenuated with higher quality snacking. In this study, we also controlled for breakfast time, and thus, late-evening snackers were experiencing longer eating windows and shorter fasting periods which may contribute towards observed unfavourable Hba1c levels. Animal studies also demonstrate that circadian disorganization impacts the intestinal microbiota [51] and the ability of fasting to beneficially modulate the gut microbiome [51, 52]; thus, late night snacking reducing the fasting window may have implications for the gut microbiome. This research supports evidence of food timing affecting circadian rhythms of organs involved in glucose metabolism [53]. Glucose tolerance exhibits diurnal variability with greater glucose response to identical oral glucose loads or meals later in the day in healthy individuals [54]. Small intervention studies in people with prediabetes suggest promising effects of limiting the eating window and restricting late-evening food consumption, i.e. time-restricted eating, on weight loss and cardiometabolic health [55]. A large proportion of people in this study were late-evening snackers indicating potential for reducing energy intake and aligning food intake with the circadian rhythms of metabolism by restricting the snacking window.

Large diversity exists in the definitions and approaches used across studies to capture snacks, whether as an eating occasion or as a specific collection of foods, contributing to conflicting evidence on the health effects of snacking. In this study, 24% of total daily energy was derived from snacks, similar to previously observed levels in other countries, including Norway (men; 17% and women; 21%) [56], Brazil (21%) [57], the UK (20%) [58] and the USA (23%) [59]. Thus, a considerable proportion of total energy is derived from snacks across countries, regardless of the definitions applied. This study also objectively measured the remainder of the diet, i.e. meal quality, to examine the impact of snacking independent of the whole day’s diet. Associations between snacking quality and main meal quality were low, highlighting the discordance between these two behaviours and their capturing of different dietary attributes suggesting that snacking is a key strategy to improve health. Further many associations were lost after adjusting for main meals and physical activity, highlighting their importance in health but the independent effect of snacking on lipemic and insulinemic responses persisted. Several studies demonstrate acute effects of high-quality snacks on postprandial metabolic responses [60, 61]. However, the positive postprandial responses associated with habitual high-quality snacking patterns have been less well documented and may be explained by protective effects of food-specific nutrients or the opportunity snacking provides to consume certain foods or nutrients not sufficiently consumed during main meals [62, 63]. Snacking on high-quality foods such as almonds has also been shown to increase alpha diversity compared to an isocaloric control [16] and increased butyrate production in adults, suggesting positive alterations to microbiota functionality [17]. Diet quality has previously been associated with the microbiome [26] but further research is required to understand habitual snacking patterns including quality and timing on gut microbiome. As eating patterns change from traditional patterns and snacking becomes ubiquitous, contributing a significant proportion to daily energy, selecting high-quality snacks is an important behavioural change that may improve long term health.

In line with previous research, our findings showed total energy intake increased with snacking frequency [64, 65]. However, the act of snacking (frequency or quantity) did not contribute to unfavourable health or weight adding to the body of research with conflicting results [20, 66, 67]. The weighted diet record approach in this study compared to habitual recall approaches often used in large epidemiologic cohorts is a possible explanation for differential association study findings. In this study, snack frequency was inversely associated with snack quality, and reduced snack quality was associated with poor cardiometabolic blood and anthropometric measures but snack frequency was not. This may be due to the larger variance observed in the quality of foods consumed by frequent snackers and supported by differences observed following stratification of individuals based on snack quality. Accounting for large inter-individual variation, we found that frequent high-quality snackers had favourable body composition compared to both non-snackers and frequent low-quality snackers. Highlighting snack choice mediates the relationship between snacking frequency and weight [20].

Strengths of this study include high-resolution diet data, weighted and checked by nutritionists in real time, to evaluate snacking intakes in a UK cohort and the densely phenotyped PREDICT cohort with postprandial metabolic responses. Limitations include the cross-sectional nature of the study which does not allow the assessment of causality owing to the uncertain temporality of the association. The associations of snacking with health outcomes may be confounded by possible under-reporting of eating frequency (that is, meal and/or snack intake) concomitant with the under-reporting of energy intake particularly by people who are overweight or living with obesity. Sample size numbers were limited when stratifying participants based on snacking frequency and quality. Finally, our data were limited to 2–4 days of logged diet data, did not have information on work versus work free days and did not permit the examination of seasonality on snacking behaviours within individuals. Compared to the average UK population, PREDICT 1 participants were older, had a lower BMI, were less likely to smoke and had a lower proportion of males (Supplementary Table 10). However, snacking intakes (% energy and frequency) were similar to previously reported surveys (24%/2.28/day vs 20%/2.55/day) (the UK and Ireland) [68]. Findings are applicable across a large proportion of the population given that the majority of people consume snacks as part of their diet habits; however, the inclusion of healthy individuals may limit generalisability to certain populations.

In conclusion, snacking behaviour may be a key diet target to ameliorate risk factors for diet-related diseases and snacking on high-quality foods earlier in the day can be part of a healthy lifestyle. However, when people have sufficient diet information, food knowledge and healthy eating intentions, the current food environment makes it difficult for them to change their snacking behaviour [69]. Public health efforts to reduce poor quality snacking must address food environments and behavioural components including food timing.

### Supplementary Information

Below is the link to the electronic supplementary material.

Supplementary Figure 1. CONSORT diagram

Supplementary Figure 2. Correlations between diet quality indices calculated using meals and snacks. uPDI, unhealthful plant diet index; oPDI, original plant-based diet index; hPDI, healthful plant-based diet index; and SDI, snack diet index

Supplementary file3 (XLSX 294 KB)

## Data Availability

Data described in the article, code book and analytic code are held with the Department of Twin Research at King’s College London and will be made available using our normal procedures overseen by the Wellcome Trust and its guidelines as part of our core funding. The application is at: https://twinsuk.ac.uk/resource s-for-researchers/access-our-data/

## Abbreviations:

SDI: Snack diet index
TG: Triglycerides
iAUC: Incremental area under the curve
HOMA-IR: Homeostatic Model Assessment for Insulin Resistance
HbA1c: Glycated haemoglobin
BMI: Body mass index
FFQ: Food frequency questionnaire
