# Supplementary figures and images for: Snack quality and snack timing are associated with cardiometabolic blood markers: the ZOE PREDICT study
Source: Eur J Nutr. 2023 Sep 15;63(1):121–33. doi: 10.1007/s00394-023-03241-6 (PMC10799113; doi:10.1007/s00394-023-03241-6)

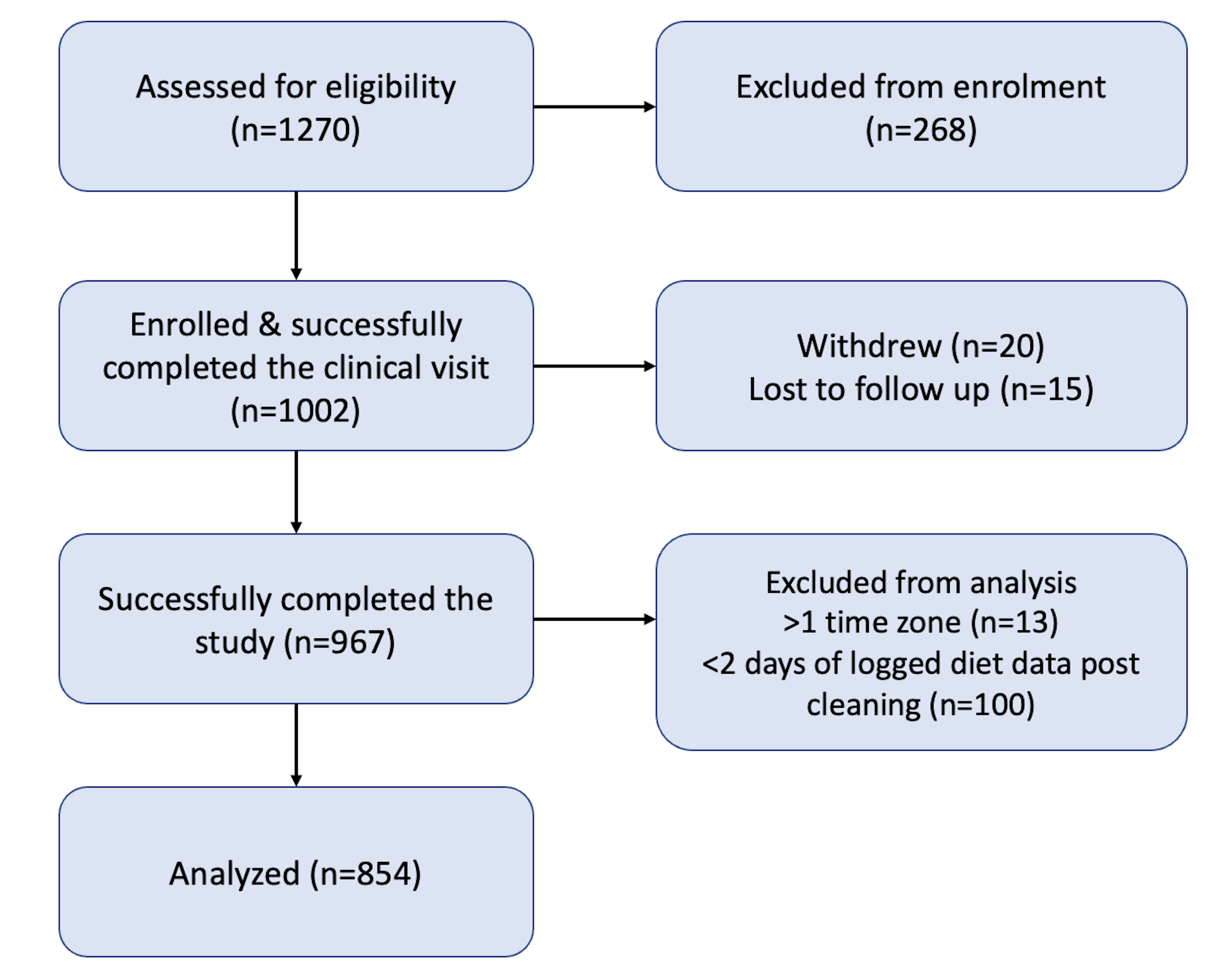

Supplement: Supplementary file 1 — Supplementary Figure 1. CONSORT diagram [file 394_2023_3241_MOESM1_ESM.tiff]

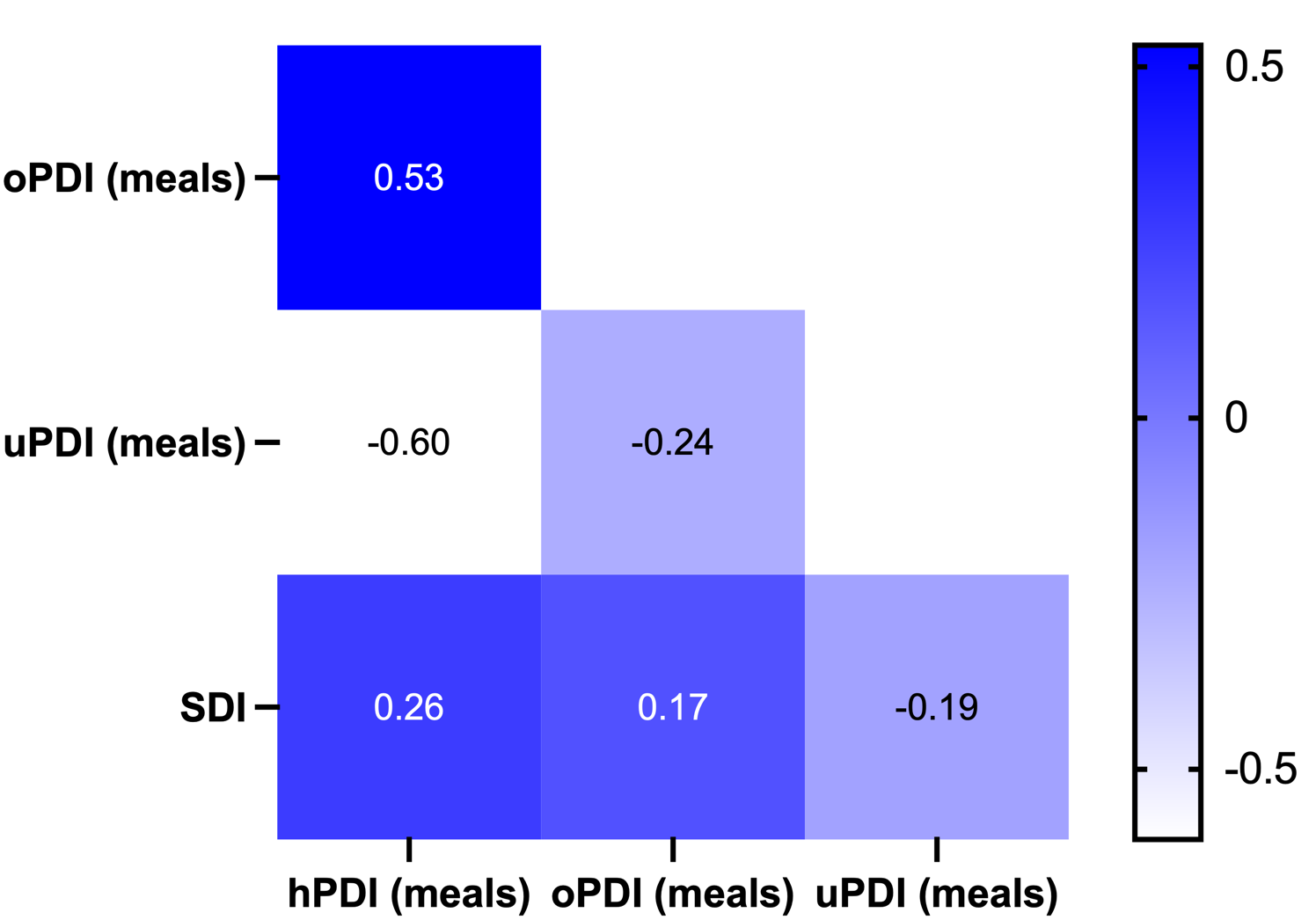

Supplement: Supplementary file 2 — Supplementary Figure 2. Correlations between diet quality indices calculated using meals and snacks. uPDI, unhealthful plant diet index; oPDI, original plant-based diet index; hPDI, healthful plant-based diet index; and SDI, snack diet index [file 394_2023_3241_MOESM2_ESM.tiff]
